# The underappreciated wrong of AIgiarism - bypass plagiarism that risks propagation of erroneous and bias content

**DOI:** 10.17179/excli2023-6435

**Published:** 2023-08-26

**Authors:** Bor Luen Tang

**Affiliations:** 1 Department of Biochemistry, Yong Loo Lin School of Medicine, National University Health System, National University of Singapore

## ⁯

We address here the use of ChatGPT in a specific aspect of research, namely academic writing, in particular its presumably common use to produce a primary or first draft of manuscripts for publication. We identify such acts as a form of plagiarism, or AIgiarism. This point is perhaps underappreciated or simply dismissed by many who deemed ChatGPT as intellectually uncreditable, either because it is not human, or because it does not qualify as an author as per widely adopted authorship guidelines. However, AIgiarism is a form of bypass plagiarism. The wrong of AIgiarism is not so much of inappropriate credit allocation, but the potential propagation of factual and interpretive errors as well as biases, which undermines knowledge acquisition and understanding. 

Mahmood and colleagues have called for caution and control in the use of ChatGPT for research (Mahmood et al., 2023[9]), a stance which we are very much in agreement with. The use of ChatGPT and other generative AI for the generation of creative content comes with clear issues associated with intellectual property rights and copyrights (Samuelson, 2023[11]). Here, we shall focus on the use of ChatGPT in academic writing and discuss an often overlooked issue with plagiarism. The notion that a human could plagiarize machine generated content is somewhat unprecedented and could easily be psychologically dismissed. As such, there are now an increasing number of articles which are admittedly (Stokel-Walker, 2023[12]), or likely generated primarily by using ChatGPT (Conroy, 2023[5]), which many would actually consider to be ethically legitimate. 

ChatGPT is a narrow AI (which is also how the chatbot describes itself (Brooks, 2023[4])) that does not have a conscious understanding of the content it generates, and does not generate academic content autonomously. GPT-3 is trained on vast amounts of text data and what it does is to machine-learn the statistical structure of natural language, and its produced output is based on a probability distribution over word sequences that allows prediction of words in a sequence (Ramponi, 2023[10]). In brief, the perception by many who use the chatbot that the party that they are conversing with is conscious of its output is an illusion. Furthermore, ChatGPT cannot take responsibility of its writings and thus could not fulfill the criteria in contemporary authorship guidelines, such as those of the International Committee of Medical Journal Editors (ICMJE) (Yeo-Teh and Tang, 2023[15]). Worryingly, almost all authors who have reported experiments with ChatGPT in writing papers to date have also observed varying degree of errors and falsification of content (Blum, 2023[3]; Conroy, 2023[5]). That Large Language Models could generate falsified content is well-known in the field of natural language processing and is termed “hallucination” (Alkaissi and McFarlane, 2023[1]; Athaluri et al., 2023[2]). In other words, ChatGPT-generated content should need to be vigorously checked for false information and inaccuracies.

Plagiarism could occur in a multitude of professional and educational settings. Here, our focus shall be on the writing of academic papers by researchers. A widely adopted definition of plagiarism in research is one stipulated by the US Office of Research Integrity - the appropriation of another person's ideas, processes, results, or words without giving appropriate credit (US Office of Research Integrity, 2022[14]). Helgesson and Eriksson have suggested that plagiarism should be understood as “someone using someone else's intellectual product (such as texts, ideas, or results), thereby implying that it is their own” (Helgesson and Eriksson, 2015[6]), with the added emphasis on the intellectual nature of the plagiarized and plagiarizables. The term “AIgiarism” is a portmanteau of the terms artificial intelligence (“AI”) and “plagiarism”^1^. AIgiarism therefore refers to acts of plagiarism committed through the use of AI. 

How does one plagiarize with AI? If an author instructs ChatGPT to generate a bibliographic list on a topic of interest for the purpose of eventually drafting a manuscript, the LLM would have been used in a search engine-like manner, which would generally not be considered plagiarism. Likewise, if one feeds ChatGPT with a written draft of a manuscript and asks that it performs language edits, ChatGPT is thereby used as a sophisticated grammar and spellchecker, and there is also no discernible plagiarism. However, if one's instruction to ChaGPT is to write a manuscript *from scratch* given a topic and various other brief specifications, we would now go into a previously uncharted territory. This is because ChatGPT, unlike any other writing or editing software or computational applications before it, is capable of generating contents that are plausible sounding, syntactically correct and semantically meaningful and rich. Furthermore, ChatGPT does this quickly and readily. If one uses this ChatGPT generated draft and submits it for publication with or without minor editing, one would have basically copied the content from ChatGPT and passing it of as one's own. Would this be plagiarism? 

We could sense the wrongness of the act described above as one being somewhat dishonest and nontransparent about the origin of the manuscript content. This is why most journals and publishers now mandate a disclosure by authors on how ChatGPT was used in manuscript writing. However, we have also alluded to the notion that ChatGPT does not qualify as an author as per contemporary authorship criteria. If so, it could not be allocated any intellectual credit like a human author, and neither would it care. This credit-based description of plagiarism would therefore appear to negate the possibility of a human plagiarizing an AI like ChatGPT. In a credit-based account on the wrong of plagiarism, the plagiarized intellect is wronged by the dishonest act of the plagiarizer, resulting in misappropriation of credit to the latter. In the case of ChatGPT, one could argue that the plagiarized is a machine and not an intellect and cannot therefore be wronged as such. If one simply discloses one's use of ChatGPT in manuscript preparation, then all would be well.

However, the wrong in AIgiarism has to be weighed from another perspective. James Stacey Taylor has recently elaborated on an “instrumental account” of plagiarism, which he posited to be the primary account of the wrong of plagiarism when this is committed by academics (Taylor, 2023[13]). The instrumental account maintains that plagiarism is wrongful because it runs counter to the purpose of academic work, which is to increase knowledge and further our understanding of science, technology, medicine or the humanities. These are ends that would be decidedly impeded by acts of plagiarism. As explained by Taylor, bypass plagiarism occurs when “*a person (C) takes another person's (B's) account of the views of a third party (A) and passes it off as though she had drawn it directly from the work of A, citing A but not B*” (Taylor, 2023[13]). Importantly, as factual and interpretive errors would likely be propagated or amplified with each round of paraphrasing and misquoting, bypass content plagiarists risk the possibility of introducing substantive errors in the content it creates based on a non-original source. 

As pointed out above, ChatGPT could be prone to “hallucinations” and the generation of fabricated or inaccurate content. Another potential issue is ChatGPT's potential to generate bias content if these are the materials on which its training is based upon. Given that we are aware of these potential pitfalls, using ChatGPT liberally to generate the primary content of an article would be a particularly reckless form of bypass plagiarism. With the above in full view, the wrong of AIgiarism is thus clear. 

However, the impact of ChatGPT in academic writing is not limited to manuscript writing. ChatGPT is not only being used to write papers, and some are also contemplating its use in reviewing papers and craft peer review reports (Hosseini and Horbach, 2023[7]). Furthermore, even the job of editors could potentially be aided or otherwise replaced by ChatGPT (Ling and Yan, 2023[8]). We could thus be facing a dreaded scenario in which scholarly papers are written by AI, reviewed by AI, read by AI and with the above processes repeated. If left unchecked, the use of ChatGPT could potentially culminate in vicious cycles that could contaminate and damage the literature in various fields with errors, inaccuracies and biases. Such cycles must not be allowed to take shape.

## Notes

1 The term first appeared on social media and can be tracked to Mike Waters' tweet (https://twitter.com/CoinWaters/status/1585453944624582656). Paul Graham has also tweeted that the rules against AIgiarism should be similar to those against plagiarism (https://twitter.com/paulg/status/1602236850105913344). 

## Conflict of interest

The author declares no conflict of interest.
